# A Pilot Study of the Relationship Between Pregnancy and Autoimmune Disease: Exploring the Mother’s Psychological Process

**DOI:** 10.3389/fpsyg.2019.01961

**Published:** 2019-08-28

**Authors:** Stefania Cataudella, Jessica Lampis, Mirian Agus, Fabiana Casula, Giovanni Monni

**Affiliations:** ^1^Department of Pedagogy, Psychology, Philosophy, Faculty of Humanities, University of Cagliari, Cagliari, Italy; ^2^Department of Prenatal Diagnosis and Fetal Therapy, Ospedale Microcitemico, Cagliari, Italy

**Keywords:** risk pregnancy, autoimmune disease, prenatal attachment, maternal representations, qualitative and quantitative analysis

## Abstract

Autoimmune disease mainly affects women in their reproductive years and has a significant impact on childbearing. Pregnancy can induce an improvement of the mother’s symptomatology in some diseases such as rheumatoid arthritis while exacerbating or having no effect on other autoimmune diseases as multiple sclerosis (Borchers et al., 2010). This uncertainty can affect the process of psychological reorganization, which leads to the achievement of a maternal identity. The quality of the mother-fetus emotional bond is considered particularly relevant for the subsequent attachment relationship and the psychological development of the infant (Ammaniti et al., 2013). In the last trimester of pregnancy, 15 women with different autoimmune diseases were interviewed using the IRMAG-R (Ammaniti and Tambelli, 2010). They also completed a battery comprising: PAI (Della Vedova et al., 2008); MAAS (Busonera et al., 2016); DAS (Gentili et al., 2002); PBI (Scinto et al., 1999); MSPSS (Prezza and Principato, 2002); DERS, (Giromini et al., 2012); CES-D (Fava, 1983); HCR-TS (Bova et al., 2012). All interviews were audiotaped, transcribed verbatim, and analyzed by Atlas.ti. The results show that women with autoimmune disease were ambivalent toward pregnancy, had high levels of depression, had difficulties in recognizing physical and psychological changes, and had difficulties in imagining the child. These are considered risk factors that could negatively affect the postnatal mother-infant relationship. These results focus on the importance of early multidisciplinary interventions that can support expectant women when they show signs of relationship difficulties with their infants prior to his/her birth.

## Introduction

Autoimmune diseases are chronic multi-system disorders characterized by organ and tissue damage caused by self-reactivity of different effectors mechanisms of the immune system, specifically antibodies and T cells. Predisposition to these diseases has been related to genetic, epigenetic, and environmental factors. Thus, autoimmune diseases mostly affect women and frequently occur during reproductive years and have implications for fertility and pregnancy (Tabarkiewicz et al., 2018). Successful pregnancy depends on early changes in the mother, which rely on modifications of the innate and adaptative immune system, inducing tolerance to the semi-allogenic fetus (Carvalheiras et al., 2012). Nevertheless, if pregnancy is planned during periods of inactive or stable disorder, the result often is giving birth to healthy full-term babies. Nonetheless, pregnancies in most autoimmune diseases are still classified as high risk because the pregnancy may have an influence on autoimmune disorder improvement or worsening and the autoimmunity increases miscarriage risks and, perinatal mortality (Borchers et al., 2010; Vengetesh et al., 2015).

Becoming a mother is one of the most central transitions in life, which requires restructuring goals, behaviors, and responsibilities to achieve a new conception of self (Kaiser et al., 2009). This transition is qualitatively influenced by her conscious and unconscious responses to it. These can be considered as biological, psychological, and psychosocial. Especially pertinent are her age, socioeconomic status, kind of autoimmune disease, and circumstances of the current pregnancy. Mental health problems such as depression and anxiety are common among people with autoimmune diseases (Boeschoten et al., 2017; Marchini et al., 2018). Autoimmune diseases, such as multiple sclerosis, can affect adjustment at both the individual and couple level. Couples facing multiple sclerosis have reported lower rates of relationship satisfaction than healthy controls (Crangle and Hart, 2017). Social support has been recognized as a protective factor for concern about maternal depressive symptoms (Harrison and Stuifbergen, 2002), predicting greater role participation, and satisfaction in mothers with multiple sclerosis (Farber et al., 2015). Trust and perceived support also are necessary for building a good patient-health care provider relationship (Bova et al., 2012). The literature shows a paucity of qualitative studies that consider the maternal experiences of women with autoimmune diseases. The focus of this issue is frequently presented from a medicalized perspective, often conducted by medical or health professionals and aimed at other professionals on how to support parents (Farber, 2000).

The woman with an autoimmune chronic disease who becomes pregnant might be more psychologically adjusted to her condition but still have fears that the pregnancy will exacerbate her disease in addition to worrying that her condition could potentially harm her baby. The occurrence of a medical illness in the expectant mother increases the complexity of her care, and it can also interfere with the woman’s ability to cope with the pregnancy and to comply adequately with the medical needs of her condition (Zager, 2009; Rasmussen et al., 2013). The increased stress experienced in high-risk pregnancies might affect the construction of the prenatal attachment (Bulbul et al., 2018). Attachment starts when a woman responds positively to pregnancy. It is known that the mother-infant relationship in the postpartum period is strongly related to the prenatal attachment (Ammaniti et al., 2013; Cataudella et al., 2016a,b).

Ehrlich (2019) argues that attachment likely plays a role in shaping immune processes. Some theoretical models underline the importance of social experiences that take place early in development that might serve as “programming” factors for the immune system. Other researchers have highlighted how ongoing social experiences in adulthood such as conflict in close relationships and ongoing stress might shape immune processes (Miller et al., 2009; Fagundes et al., 2013).

These premises underline the need for attention to factors that can positively or negatively affect the transition to motherhood for women with autoimmune diseases. The central aim of our pilot study was to explore psychological dynamics during pregnancy as well as the possibility of evidencing vulnerabilities and risk factors that could negatively interfere with the establishment of a bond between a mother and her baby.

## Methods

### Participants

Fifteen Italian women were contacted during their follow-up visits to the Department of Prenatal Diagnosis and Fetal Therapy “Ospedale Microcitemico” in Cagliari (Italy). They have a mean age of 36.4 years (SD = 5.4); their mean gestational age was 29.6 weeks (SD = 5.6).

Sociodemographic characteristics and pregnancy-related variables are reported in Table 1.

**TABLE 1:** Sociodemographic characteristics and pregnancy-related variables.

**Variable**	**Frequency**
**Age group**	
28–35 years	7
36–46 years	8
**Educational level**	
Middle school	1
High school	2
University degree	9
Postgraduate	3
**Marital status**	
Married	12
Cohabiting	2
Engaged	1
**Parity**	
Primiparae	9
Multiparae	6
**Previous miscarriage(s)**	
Yes	5
No	10
**Pregnancy planning**	
Planned	11
Unplanned	4
**Sex of the baby**	
Male	6
Female	8
Missing	1
**Types of autoimmune diseases**	
Mixed connective tissue diseases	1
Atopic dermatitis	1
Type 1 diabetes	2
Psoriasis	1
Autoimmune thyroiditis	5
Systemic lupus erythematosus	1
Multiple sclerosis	2
Autoimmune thyroiditis + Type 1 diabetes	1
Autoimmune thyroiditis + Alopecia Areata	1
**Time from diagnosis**	
<5 years	1
>5 years	11
Missing	3

### Procedure

All expectant women were contacted and interviewed by psychologists skilled in administering all investigation instruments. The presence of autoimmune disease constituted the inclusion criteria in the study. All pregnant women voluntarily accepted to participate in the research; they signed the format of informed consent and were interviewed. Then, they completed some self-report questionnaires, fulfilled between the second and third trimester of pregnancy. The study is still ongoing, and it includes a follow-up 3 months after birth. In this paper we illustrate the results of the sample at T1 (during pregnancy).

The study was accepted by the Ethics Committee at the University of Cagliari - Italy (referring to the Department of Pedagogy, Psychology, Philosophy).

### Measures

#### Questionnaire on Sociodemographic Characteristics and Pregnancy Related Variables

This questionnaire is devised *ad hoc* to collect some relevant information (e.g., age, educational level, gestational age, parity, pregnancy planning, marital status, type of autoimmune disease and, time of diagnosis).

#### Interview of Maternal Representations During Pregnancy-Revised Version (IRMAG-R; Ammaniti and Tambelli, 2010)

This interview was characterized by 41 questions; they assessed in detail the effect of traumatic past and/or recent experiences, furthermore the occurrence of mother’s preoccupations and/or disproportionate fears regarding the woman or the baby. The interview encourages the woman’s description of her experience regarding her gestation and the process of becoming mother, investigating the mental representations of this woman as a mother and of her expected baby.

#### Prenatal Attachment Inventory (PAI; Muller, 1993; Della Vedova et al., 2008; Busonera et al., 2017)

This is designed to measure prenatal attachment according to Muller’s (1993) definition. It is composed of 21 items assessed by a Likert scale (ranging from 1 - *almost never -* to 4 - *almost always*). High scores indicate a high grade of prenatal attachment. The reported internal reliability values of Alpha vary from α = 0.81 to α = 0.93.

#### Maternal Antenatal Attachment Scale (MAAS; Condon, 1993; Busonera et al., 2016)

This measure assesses *Quality of attachment* (constituted by 11 items) and *Intensity of preoccupation* (defined by 8 items). The high global score denotes a high level of attachment to the unborn baby. The values of Cronbach’s α were reported ranging between 0.69 and 0.87.

#### Dyadic Adjustment Scale (DAS; Spanier, 1976; Gentili et al., 2002)

This instrument (characterized by 32 items) consisted of four dimensions: Affective expression (4 items); Cohesion (5 items); Consensus (13 items); Satisfaction (10 items). Reported internal consistency ranges from 0.73 to 0.96.

#### Parental Bonding Instrument (PBI; Parker et al., 1979; Scinto et al., 1999)

It assesses the view that adult have of the parenting style of their mothers and fathers. It is composed of 25 items for the mother and 25 items for the father, assessed by Likert scale (ranging from 0 = *very unlike* to 3 = *very like*). The parenting style is evaluated in terms of *care* (12 items) and *control* (13 items). Both the original and italian versions of the instrument showed good internal consistency (from 0.83 to 0.91).

#### Multidimensional Scale of Perceived Social Support (MSPSS; Zimet et al., 1988; Prezza and Principato, 2002)

This measure (characterized by 12 items) assesses the appropriateness of support from some figures: family, friends, and a significant other. All responses are rated on a 7-point Likert scale and high scores are related with the perception of high levels of social support. This instrument showed good indices of reliability (Alphas from 0.81 to 0.98).

#### Center for Epidemiologic Studies Depression Scale (CES-D; Radloff, 1977; Fava, 1983)

This test comprises 20 items with responses assessed by a 4-point Likert-type scale. The total scores vary from 0 to 60 (when high scores designate a strong depressive symptomatology). The CES-D values of reliability Alpha coefficients varied from 0.85 to 0.95.

#### Difficulties Emotional Regulation Scale (DERS; Gratz and Roemer, 2004; Giromini et al., 2012)

It assesses clinically significant difficulties in the process of emotion regulation. The 36 items referred to six dimensions: Difficulties Engaging in Goal-Directed Behavior (5 items); Impulse Control Difficulties (6 items); Lack of Emotional Clarity (5 items); Lack of Emotional Awareness (6 items); Limited Access to Emotion Regulation Strategies (8 items); No acceptance of Emotional Responses (6 items). Questions are assessed by a Likert scale (ranging from 1 - *almost never -* to 5 - *almost always*). The authors reported a good internal consistency (α = 0.93).

#### Health Care Relationship-Trust Scale Revised (HCR-TS; Bova et al., 2012)

This 13-item scale assesses patients’ levels of trust in their healthiness care provider. Items are evaluated from 0 to 4. Total scores have a possible range of 0–52 (when higher scores indicate greater levels of trust). Cronbach’s alpha was 0.96. This instrument did not have a validation study for the Italian version; therefore, the results were prudently evaluated referring to the original normative sample.

### Data Analysis

In order to deepen the complexity of the matter, we applied a mixed methods approach. Specifically, we chose to combine qualitative and quantitative data, emphasizing the convergence of suggestions deriving from different methods that measure related unobserved construct. The subsequent “triangulation” among dissimilar methods and data allows for a full investigation of relevant experimental dimensions (Creswell and Creswell, 2017).

In relation to the application of a qualitative approach, in these preliminary analyzes, we did not use the IRMAG-R coding system provided by the authors, but the transcripts were analyzed applying a methodological approach broadly inspired to the general principles of Grounded Theory. Specifically, in relation to the qualitative analysis of the transcripts, we applied a process of interpretative reconstruction of the information, based on cyclical comparisons of data. These recurrent assessments have supported in this clinical sample a constructive dynamic identification of encoding steps (Cicognani, 2002). This qualitative approach allows us to catch useful clinical insights to understand emotional and/or psychic dynamics activated during pregnancy. In particular, this approach started from the attribution of codes, a progressive reassembly of data, together with a detailed analysis of the same data, to achieve an increasing level of abstraction. We identified 48 *Codes* grouped into six larger *Families* defined as: (1) perception of maternal identity, (2) creation of a mental space for the baby, (3) perception of couple changes, (4) association between pregnancy and autoimmune disease, (5) occurrence of narrative’s contradictions, (6) emotions and fears.

Each *Family* was divided into *functional* (*N* = 27/48 codes) and *dysfunctional* (*N* = 21/48 codes) *aspects* (Table 2). Following the recommendations given by Corbin and Strauss (2008), the data were examined and debated by several researchers. All interviews were recorded, transcribed verbatim, and analyzed using the software Atlas.ti (release 7.5). All interviews were coded by two researchers to ensure multiple perspectives on the data (agreement = 82%).

**TABLE 2:** Description of FAMILIES; frequencies and percentage of functional and dysfunctional codes.

**Family codes N Functional – Dysfunctional codes (e.g., functional and dysfunctional aspects)**	**N Functional Frequency (%)**	**N Dysfunctional** **Frequency (%)**	**Total**
**(1) Perception of maternal identity 5 - 4** (*functional*: code 1. In her narrative, the woman is able to express her own identity as mother; *dysfunctional*: code 2. In her narrative, the woman is unable to express her own identity as mother)	88 (85.5%)	15 (14.5%)	103
**(2) Creation of a mental space for the baby 10 - 7** (*functional*: code 10. The woman describes fetus’s movements and she describes these movements as traits of the baby; *dysfunctional*: code 15. The woman refers to the baby using neutral terms such as “the baby” and does not use nicknames or the chosen name)	166 (28.2%)	421 (71.8%)	587
**(3) Perception of couple changes 8 - 3** (*functional*: code 31. The woman refers to changes in the relationship with the partner such as greater intimacy; *dysfunctional*: code 35. The woman says that nothing has changed in her relationship with her partner during her pregnancy)	48 (73.8%)	17 (26.2%)	65
**(4) Association pregnancy-autoimmune disease 1 - 2** *(functional*: code 25. The woman spontaneously reflects on the possible interactions between her own autoimmune disease and pregnancy; *dysfunctional*: code 27. In her narrative, the woman never refers to her own autoimmune disease)	17 (65.3%)	9 (34.7%)	26
**(5) Occurrence of narrative contradictions 0 - 4** (*dysfunctional*: code 45. In the interview with respect to the same subject, the woman expresses thoughts in contrast with each other)	/	100 (100%)	100
**(6) Emotions and fears 3 - 1** (*functional*: code 42. The woman talks about her positive and negative emotions. *dysfunctional*: code 39. The woman refers irrational fears such as “*I am afraid that with the fetal movements, the membrane will break”*)	480 (99%)	4 (1%)	484
**N Tot Functional codes: 27/48** **N Tot Dysfunctional codes: 21/48**			

Regarding the quantitative approach, we analyzed the outcomes of our participants in the validated questionnaires, comparing their scores with the normative samples’ means. Specifically, we applied the Student’s *t* to evaluate if the participants’ means were different from the normative sample.

## Results

**FIGURE 1:**
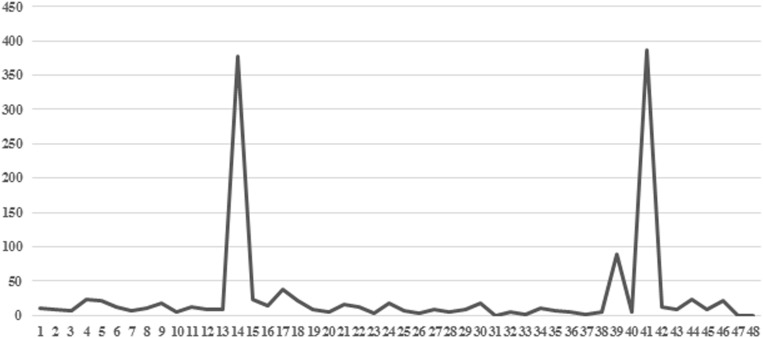
Frequency of 48 codes for 15 interviews. In the axis of the abscissas are the codes; in the axis of the ordinates are the frequencies of the codes.

In the first phase of work, we evaluated the occurrences of *codes* in the interviews. The most frequent *codes* were (Figure 1): *Code* 15 (*Dysfunctional*) *No name for the baby* (*N* = 357): the mother refers to the baby using neutral terms such as “the baby” and does not use nicknames or the name chosen; *Code* 40 (*Functional*) *Fears* (*N* = 88): the mother reports fears related or not related to pregnancy which appear to be “natural” in this phase of life (e.g., the fear of delivery); *Code* 42 (*Functional*) *Emotions* (*N* = 387): the mother talks about her positive and negative emotions.

The most frequent *Functional Family Codes* were: (1) Perception of maternal identity (85.5%); (3) Perception of couple changes (73.8%); (4) Associations pregnancy-autoimmune disease (65.3%); (6) Emotions and fears (99%).

The most frequent *Dysfunctional Family Codes* were: (2) Creation of a mental space for the baby (71.8%); (5) Occurrence of narrative contradictions (100%) (Table 2).

Descriptive statistics of measures and statistical comparison with normative samples are reported in Table 3. It is highlighted a significant difference (*df* = 14; *p* < 0.01) between the score of our participants and the mean scores in normative samples in relation to the following scales: DAS Dyadic Cohesion (*t* = 5.27); PBI Paternal Care (*t* = −6.58) and Maternal Care (*t* = −8.60); MSPSS Tot (*t* = −5.33), Family (*t* = −3.33), Friends (*t* = −6.77) and Significant Other (*t* = −3.80); CES-D (*t* = 7.40); DERS Goals (*t* = 39.10); HCR-TS D (*t* = −6.44).

**TABLE 3:** Descriptive statistics of measures – statistical comparison with normative sample.

**Scale**	**Mean (sd)**	**Mean (sd)**	**Student’s *t* (df = 14)**
			
	**Participants**	**Normative sample**	
**PAI**	60.7 (9.1)	60.9 (9.2)	−0.07
**MAAS**	76.6 (5.3)	78.6 (5.6)	−1.93
**DAS**		
Tot	115 (30.3)	115.7 (21.6)	−0.13
Dyadic consensus	53.3 (6.0)	51.6 (10.1)	1.05
Dyadic satisfaction	38.2 (8.9)	37.7 (7.7)	0.21
Affectional expression	9.47 (2.5)	9.8 (2.4)	−0.51
Dyadic cohesion	18.5 (2.7)	14.6 (5.2)	5.27^∗∗^
**PBI**		
Paternal care	19.4 (3.3)	25.1 (8.1)	−6.58^∗^^∗^
Paternal overprotection	13.2 (5.7)	13.5 (8.0)	−0.23
Maternal care	21.1 (2.9)	27.6 (7.5)	−8.60^∗^^∗^
Maternal overprotection	14.9 (6.5)	16.3 (8.6)	−0.85
**MSPSS**		
Tot	5.16 (0.6)	6.0 (0.8)	−5.33^∗∗^
Family	5.05 (0.9)	5.9 (1.2)	−3.33^∗∗^
Friends	5.11 (0.5)	6.1 (0.8)	−6.77^∗∗^
Significant other	5.33 (0.7)	6.0 (1.1)	−3.80^∗∗^
**CES-D**	32.5 (9.7)	12.9 (7.8)	7.40^∗∗^
**DERS**		
Tot	71.7 (17.9)	73.6 (16.1)	−0.40
Non-accept	12.9 (4.1)	11.2 (3.7)	1.54
Goals	38.9 (2.5)	13.6 (4.4)	39.10^∗^^∗^
Impulse	10.3 (3.5)	11.6 (3.4)	−1.46
Awareness	13.5 (4.5)	14.0 (3.7)	−0.45
Strategies	12.9 (4.0)	14.1 (5.0)	−1.12
Clarity	9.2 (3.3)	9.2 (2.6)	0.00
**HCR-TS**	39.1 (9.7)	55.3 (7.1)	−6.44^∗∗^

*^∗∗^*p* < 0.01.*

## Discussion

The results of our study showed that the participants did not have socio-demographic characteristics that could be considered risk variables (see Table 1). Nevertheless, the results from the qualitative and quantitative analyses highlighted some vulnerabilities that could have negative effects on bonding between mother and fetus/child. Qualitative analysis of interviews showed that these women focus in their narratives on greater intimacy with the partner and give less space to the representation of the baby and the construction of the bond with him/her. Furthermore, transcripts are often characterized by statements that are contradicted during the interview and that can refer to each topic addressed. The women did not always spontaneously reflect on the possible interactions between their own autoimmune disease and their pregnancy. Women give much space to the verbalization of emotions, both positive and negative, but the emotions appear more centered on themselves and less on the baby. Moreover, emotions often express contradictory aspects (e.g., a mother with autoimmune thyroid disease says at the beginning of the interview: “I was happy at the news of the pregnancy” and in another part of the interview she claims to have been very ambivalent toward pregnancy although the pregnancy itself was planned; a mother with multiple sclerosis says: “I never thought of the disease as a problem, I never counted a relapse, I didn’t want the disease to limit me”; a mother with type 1 diabetes says: “I never imagine the baby; I feels that the baby moves a lot but I don’t know what could stimulate these movements; I didn’t prepare anything at home for his/her birth” and then she says: “I think I’ll be a careful mother”; a mother with multiple sclerosis says “I’m afraid the baby suffers when he moves too much, maybe he moves a lot because he can suffocate. I have feelings of guilt because I drink little so I am afraid that the amniotic fluid will dry up”; a mother with autoimmune thyroid disease says “our sexual activity has increased a lot, we really want to stay close, the feeling has increased. There is more sexual desire but recently it is difficult for this belly that disturbs”). These aspects seem to converge with the   results   of   the   quantitative   analysis   (see Table 3).

The women showed a difficulty in regulating emotions (Goals subscale of the DERS “difficulties engaging in goal-directed behavior when emotionally aroused”: 38.9 vs. 13.6; CES-D: 32.5 vs. 12.9) and low perceived social support (MSPSS total: 5.16 vs. 6.0). Women also referred memories of low maternal and paternal care (PBI paternal care: 19.4 vs. 25.1; maternal care: 21.1 vs. 27.6) and low trust in the health care provider (HCR-TS: 39.1 vs. 55.3). However, the women reported a high level of attachment to the fetus and a good marital relationship characterized by a high score on the subscale of dyadic cohesion (18.5 vs. 14.6) that indicates the degree of closeness and shared activities experienced by the couple. Despite, the high level of attachment to fetus it seems that in the process of psychic reorganization functional to the construction of the maternal role, these women show a difficulty in holding the baby in their mind almost comparable to the difficulty of their immune system receiving the fetus. This probably leads to the emergence of emotional states that, instead of reorganizing the emotional experience toward the construction of the definition of the parental role and the representation of the other, lead to the emergence of depressive symptoms and low trust in the health care providers and in perceived social support from their network of relationships. The characteristics of the narrative seem to approach the group identified by Ammaniti et al. (2013) as Not Integrated/Ambivalent characterized by confused and contradictory representations, which limit the possibility of a coherent narration of the mother’s personal experience while the affective investment is quite high. A subsequent analysis of these interviews through the IRMAG coding system will allow us to verify these data. The literature (Fava Vizziello et al., 1993; Stern, 1995; Rasmussen et al., 2013) highlighted that the difficulty to organize representation of the baby, the occurrence of depression and/or low social support, and memories of low maternal and paternal care can lead to potentially disturbed mother-infant relationships. These preliminary results give interesting insights on the possible impact of autoimmune disease on the redefinition of maternal psychic equilibrium. With the addition of a medical condition, the pregnancy requires constant attention to the woman’s emotional and physical well-being. The woman’s emotional response can also be affected by changes necessitated by the high-risk condition (e.g., frequent examinations to monitor fetal growth). An ambivalent attitude during pregnancy can predict a higher probability of difficulties in maternal caregiving. Sometimes these complicating emotional and psychosocial factors are overlooked because the attention only focuses on medical conditions. Recognizing mothers’ vulnerabilities during pregnancy is important because it underlines the need for a supportive intervention during this period. A good working relationship between the woman and the medical staff is essential so that the woman receives all information about her autoimmune disease and her baby’s health in the most appropriate manner. The maternal-fetal immunological interrelationship is an important association between two different individuals. Psychoneuroimmunology has brought forth remarkable insights that highlight how the relational world can “get under the skin” to influence immune, neural, and neuroendocrine processes in ways that might have consequences for later health (Ehrlich, 2019). It’s important to reflect on the complexity of the mind-body relationship and on the role of physical and psychic defense mechanisms in the processes of change. The relationship between pregnancy and autoimmune disease refers to the relationship between the role of care and the role of protection. Often pregnancy, with its physiological transformations, acts on the woman’s immune system, protecting the woman from the symptoms of the disease. What, then, is the baby’s role in the mother’s mind? The presence of a medical condition, which can compromise the physical health of mother and baby, often obligates both the mother and the care setting to pay attention to the body of the woman and the fetus/child, neglecting psychological needs, thus supporting a possible mind-body scission. Understanding these aspects is relevant both for supporting mothers and for professionals who follow these women in the perinatal period.

Limitations of the present study are that it included a limited sample size and the heterogeneity of autoimmune diseases with possible different effects on pregnancy. This is due to the difficulty of recruiting the sample in this population. Furthermore, these features of the sample size do not allow to apply more sophisticated statistical analyses. An additional limit of this work might be related to the absence of the control group in this phase of the work. A control group will be used in the next steps of our research, which is actually in progress.

## Data Availability

The datasets generated for this study are available on request to the corresponding author.

## Ethics Statement

The studies involving human participants were reviewed and approved by the Ethics Committee of the Department of Pedagogy, Psychology, Philosophy at the University of Cagliari (Italy). The patients/participants provided their written informed consent to participate in this study.

## Author Contributions

SC, JL, and MA contributed equally to the theoretical and empirical aspects of the study, and wrote the final version of the manuscript. FC contributed to the collection and analysis of data. GM contributed in critically revising the manuscript for important intellectual content.

## Conflict of Interest Statement

The authors declare that the research was conducted in the absence of any commercial or financial relationships that could be construed as a potential conflict of interest.
